# Recurrence of malignant insulinoma in the context of multiple endocrine neoplasia type 1: a case report

**DOI:** 10.1093/jscr/rjaf335

**Published:** 2025-05-22

**Authors:** Marcela Palomo, Ximena Rivera, Isabella Santamarina

**Affiliations:** Department of Medical Research, Universidad Francisco Marroquin, 6ta calle final zona 10, Guatemala City 01010, Guatemala; Department of Medical Research, Universidad Francisco Marroquin, 6ta calle final zona 10, Guatemala City 01010, Guatemala; Department of Medical Research, Universidad Francisco Marroquin, 6ta calle final zona 10, Guatemala City 01010, Guatemala

**Keywords:** malignant insulinoma, multiple endocrine neoplasia type 1, pancreatic neuroendocrine tumor, recurrent insulinoma

## Abstract

Insulinomas are rare pancreatic neuroendocrine tumors, typically benign, but in the context of multiple endocrine neoplasia type 1 (MEN1), they can be malignant and recurrent. This case report presents a 58-year-old man with MEN1 who initially underwent partial pancreatectomy for a malignant insulinoma in the pancreatic tail in 2019. Despite recurrence in 2021, the patient declined surgery; however, in 2025, he presented with hypoglycemia and imaging revealed a 3-cm mass in the pancreatic head. He underwent a Whipple procedure, with histopathological analysis confirming the recurrent insulinoma. This case highlights the clinical challenge of managing malignant insulinomas in MEN1, emphasizing the importance of long-term follow-up and staged surgical interventions due to the high risk of recurrence.

## Introduction

Insulinomas are rare pancreatic neuroendocrine tumors (PNETs) that are typically benign, but their behavior can differ when associated with multiple endocrine neoplasia type 1 (MEN1). In patients with MEN1, insulinomas have a higher likelihood of being malignant and recurrent, posing significant clinical challenges. These tumors are commonly solitary and smaller than 2 cm, but in the setting of MEN1, they may present with more aggressive characteristics, including local recurrence and metastasis. We present the case of a 58-year-old male with MEN1 who developed a malignant insulinoma in the pancreatic tail, which recurred in the pancreatic head, highlighting the complexities of managing insulinomas in the context of MEN1. This case emphasizes the importance of long-term surveillance and a staged surgical approach to optimize outcomes and address the potential for recurrence.

## Case report

A 58-year-old man presented to the emergency department in January 2025 with dizziness, generalized weakness, and diaphoresis. His symptoms were confirmed to be due to hypoglycemia, with documented blood glucose levels as low as 56 mg/dl.

In 2019, in the context of MEN1, he was diagnosed with an insulinoma in the pancreatic tail and underwent partial pancreatectomy. The diagnosis was confirmed by biopsy, which reported a well-differentiated PNET with vascular invasion and a high proliferation index, suggestive of malignant insulinoma. In 2021, he experienced a recurrence of the insulinoma. However, at that time, the patient declined surgical treatment.

Among other endocrine manifestations of MEN1, a somatotropin-lactotropin macroadenoma in the pituitary gland and bilateral parathyroid adenomas with hyperparathyroidism were documented. Additionally, he had Stage V chronic kidney disease and was undergoing hemodialysis three times per week. His family history was notable for a paternal diagnosis of a macroadenoma and pancreatic cancer.

**Figure 1:**
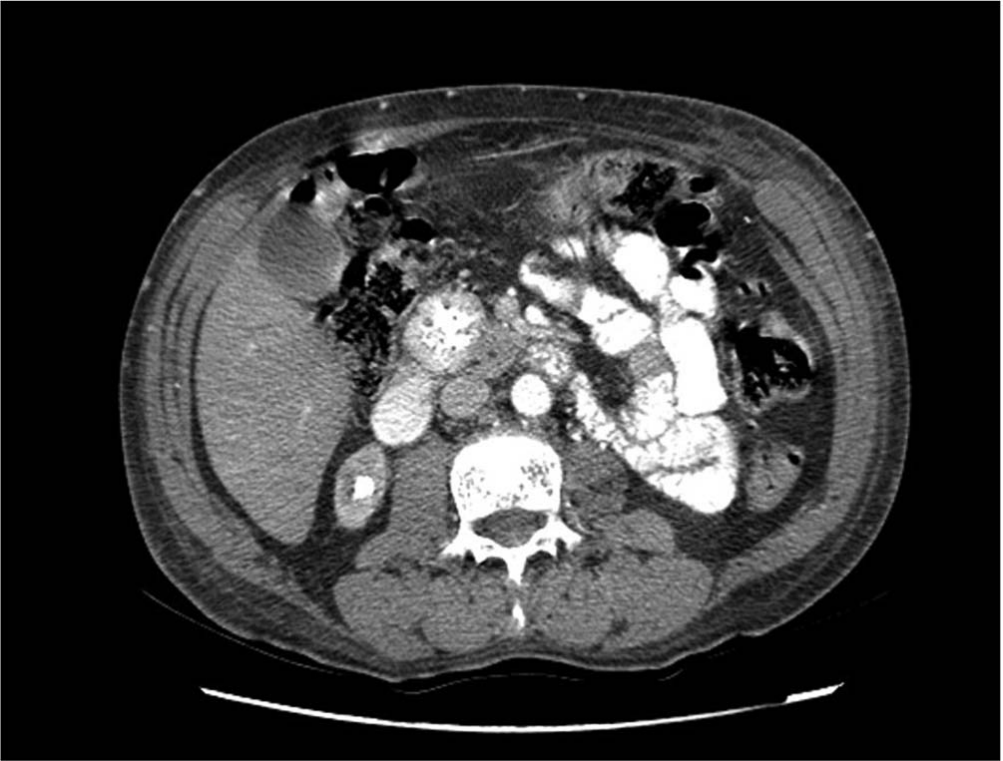
Triphasic computed tomography of the pancreas, arterial phase, showing a tumor in the head of the pancreas.

**Figure 2:**
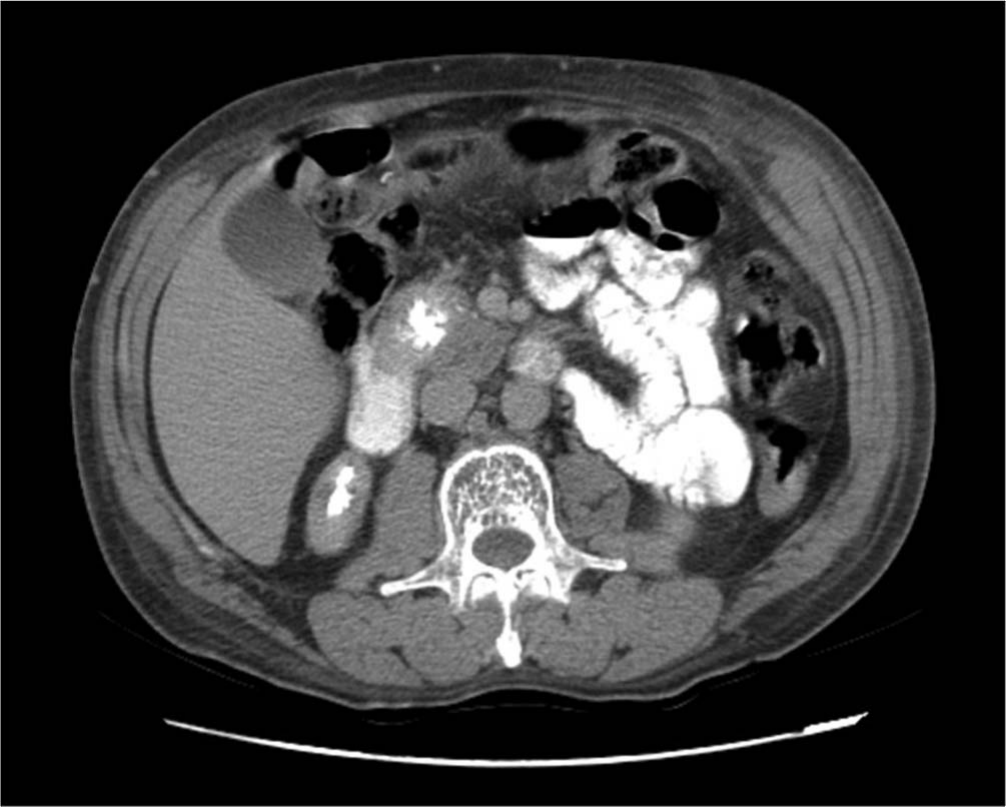
Triphasic computed tomography of the pancreas, late phase, showing a tumor in the head of the pancreas.

**Figure 3:**
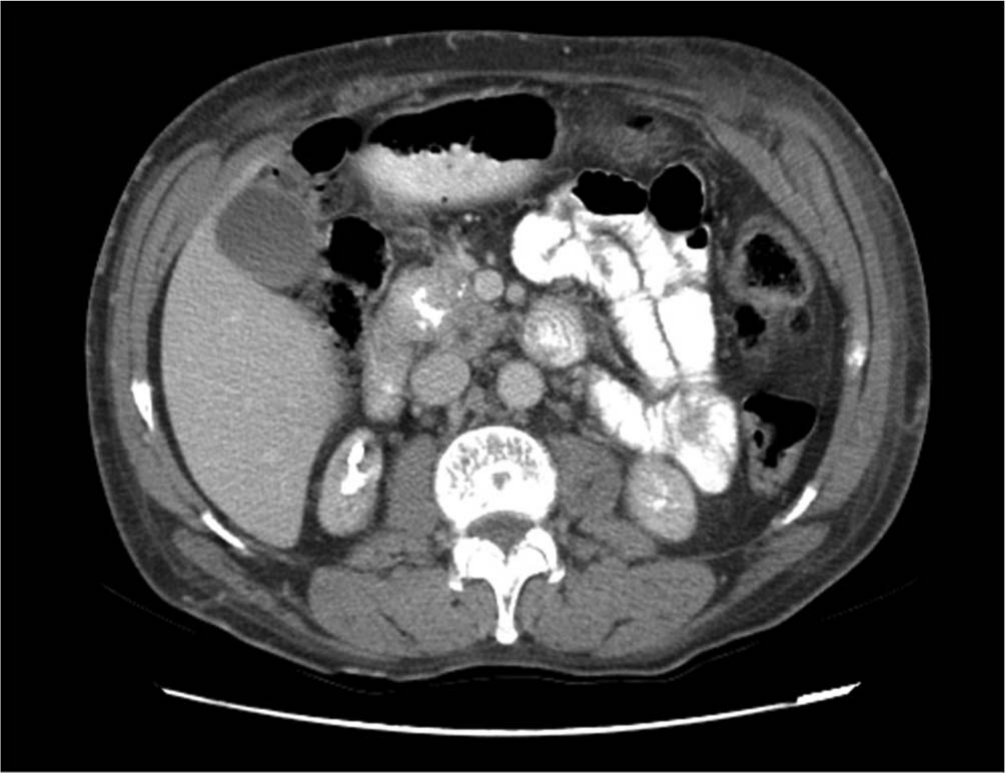
Triphasic computed tomography of the pancreas, portal phase, showing a tumor in the head of the pancreas.

Upon admission in January 2025, the patient presented with altered mental status, dehydration, and hypoglycemia. The recurrence of hypoglycemic episodes was confirmed by laboratory studies and imaging, which identified a well-defined 3 cm mass in the pancreatic head on triphasic computed tomography (Figs 1–3). Given the diagnosis of recurrent malignant insulinoma and the possibility of resection, a surgical approach was chosen.

**Figure 4:**
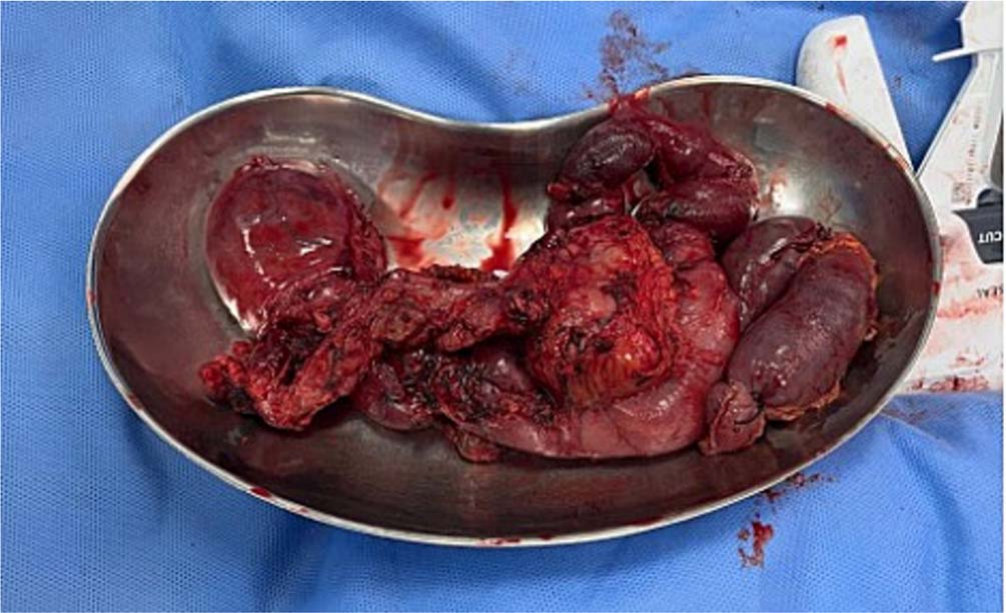
Surgical specimen.

**Figure 5:**
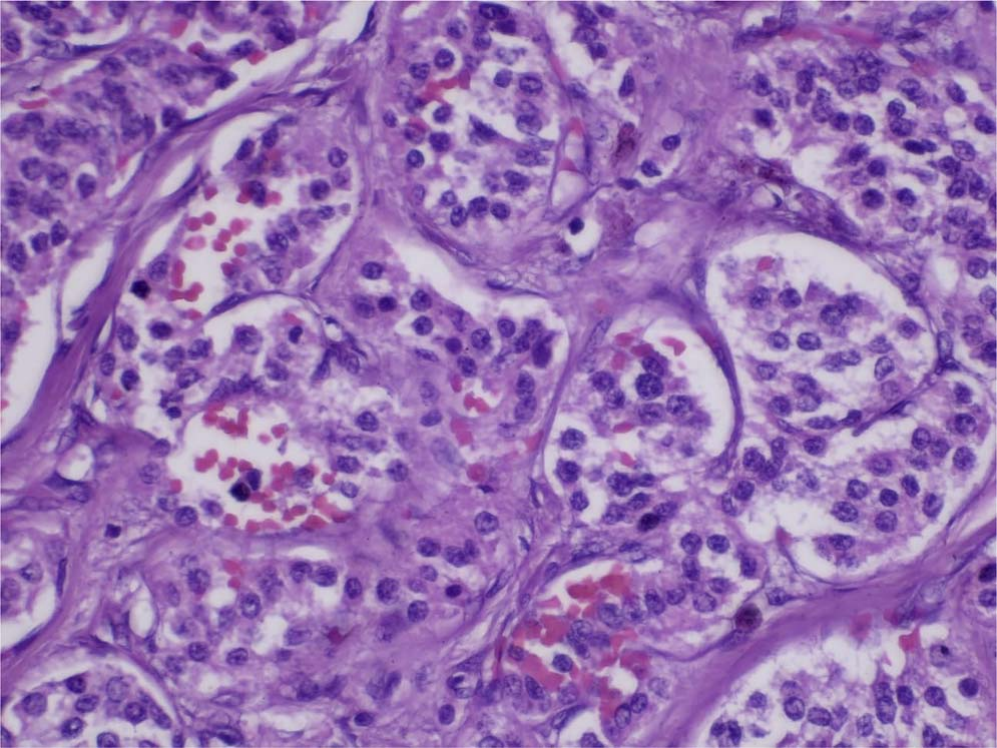
Hematoxylin and eosin (H&E) stain, 400× magnification. Tumor cells arranged in nested and trabecular patterns with round, hyperchromatic nuclei, and finely granular chromatin.

**Figure 6:**
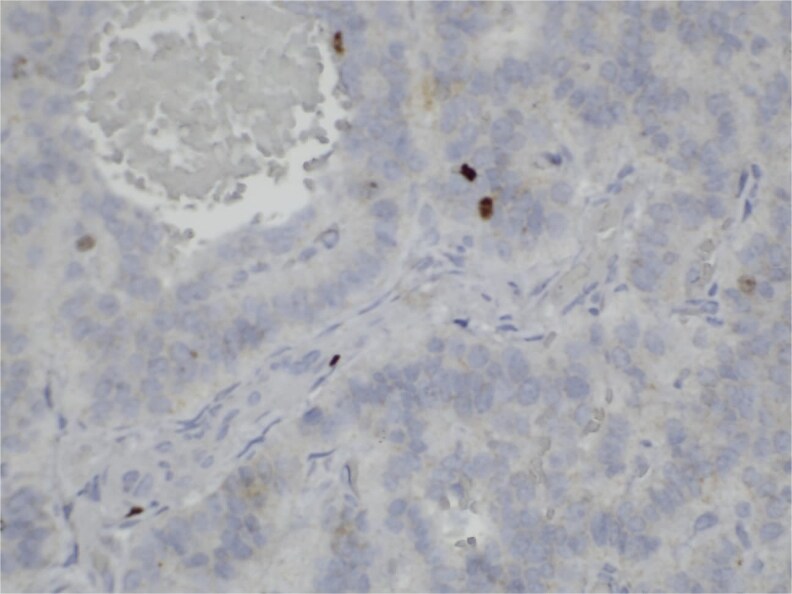
Immunohistochemistry for Ki-67. Scattered nuclear staining is observed, indicating a low proliferative index (2%).

**Figure 7:**
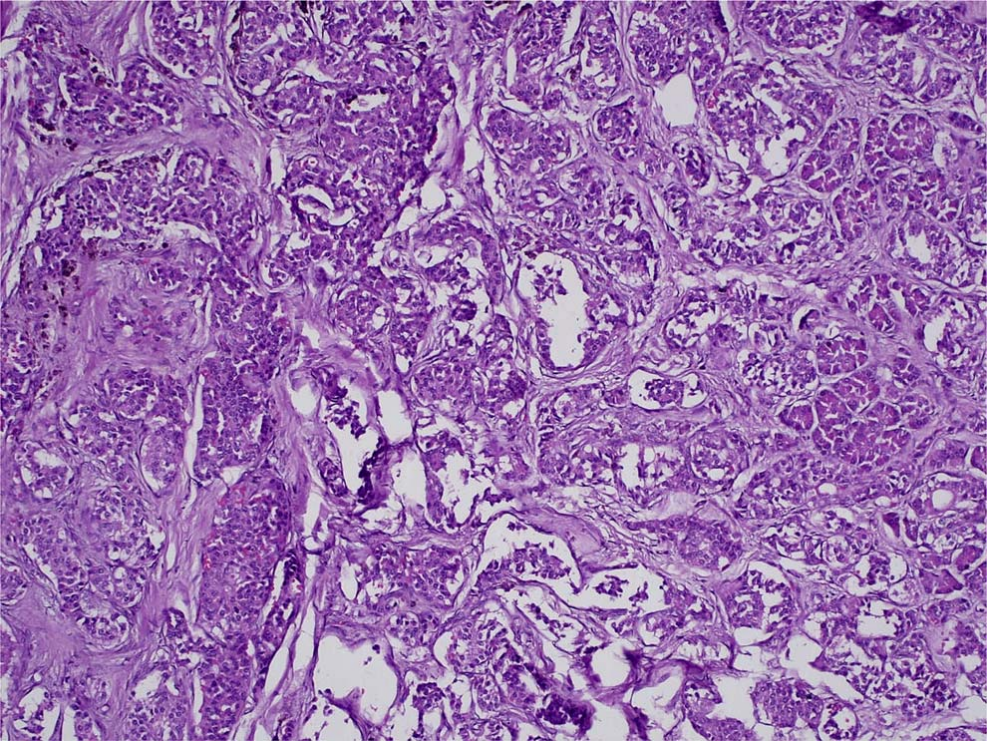
H&E stain, 100× magnification. Diffuse infiltration of pancreatic parenchyma by tumor cells with desmoplastic stroma.

**Figure 8:**
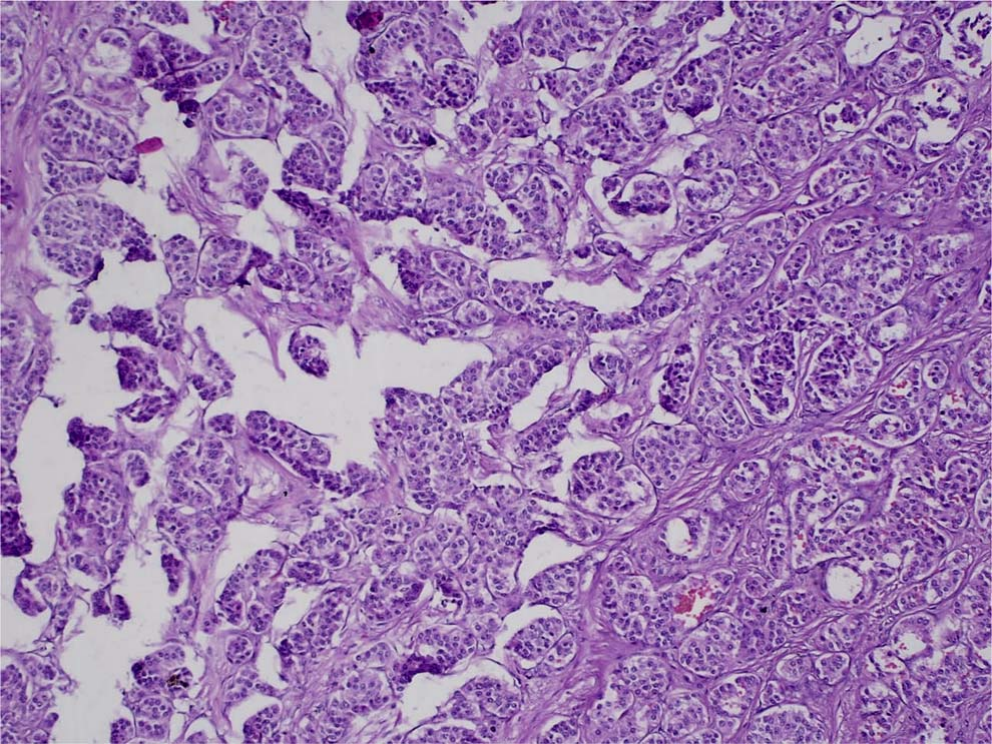
H&E stain, 100× magnification. Irregular clusters of neoplastic cells with vascular invasion.

**Figure 9:**
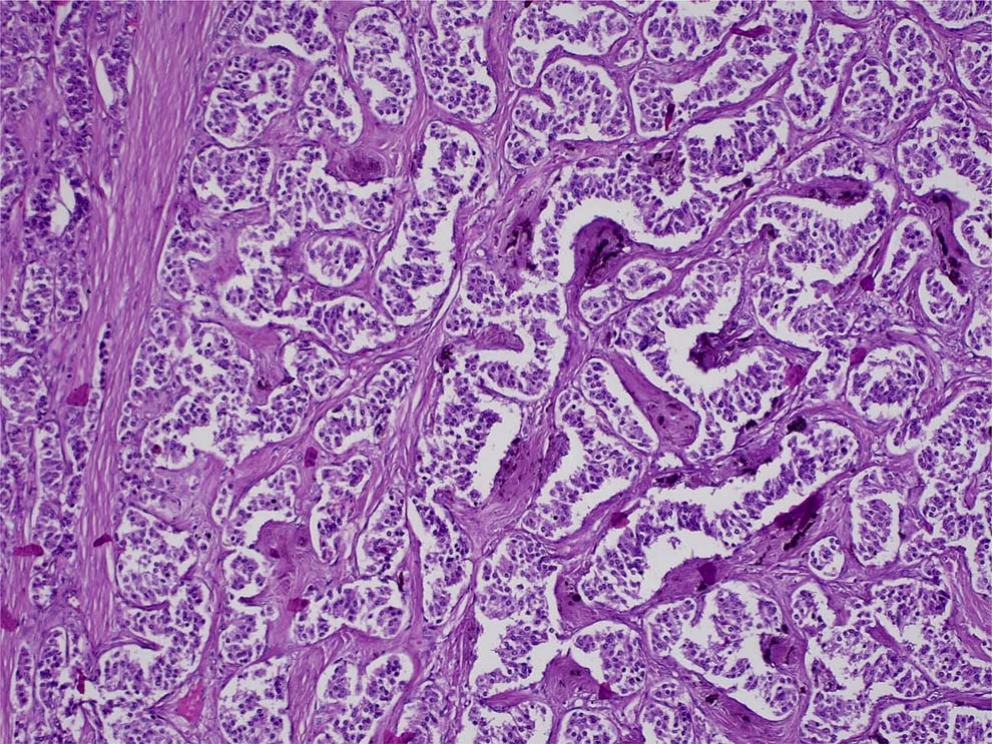
H&E stain, 100× magnification. Papillary and trabecular architecture with minimal atypia and characteristic “salt and pepper” chromatin.

On 27 January 2025, the patient underwent a Whipple procedure (pancreaticoduodenectomy). The surgery included cephalic pancreatectomy with reconstruction through hepaticojejunostomy and gastrojejunostomy in a Roux-en-Y configuration. Gross pathology revealed a firm, white-yellow, indurated mass in the pancreatic head measuring 3.7 × 3.5 × 3 cm with irregular borders and central hardness (Fig. 4). Histopathological analysis confirmed a Grade 1 neuroendocrine tumor (insulinoma), with a Ki-67 index of 2%. The tumor showed direct extension to adjacent pancreatic tissue, the duodenal muscularis propria, the adventitia, and the muscularis of the common bile duct. Five peripancreatic lymph nodes were dissected, three of which were positive for metastases (Figs 5–9).

Postoperatively, the patient was transferred to the intensive care unit, requiring vasoactive agents, supplemental oxygen, and developed acute pulmonary edema secondary to fluid resuscitation and transfusions. He experienced episodes of hyperglycemia, which were managed with insulin therapy. Hemodialysis was continued as per his prior regimen. Three days after surgery, the patient was transferred to the general ward, no longer requiring oxygen support or vasoactive medications.

## Discussion

Insulinomas are rare PNETs with an annual incidence of one to three cases per 1 000 000 patients [1]. Approximately 90% of insulinomas are benign, 90% are solitary, and 90% measure <2 cm [2]. Most insulinomas are sporadic, and only 5%–10% occur in association with MEN1 [3, 4]. The tumor in our patient was a malignant, recurrent, solitary insulinoma measuring 3 cm, occurring in the context of MEN1.

PNETs are classified into those associated with functional syndromes and those without associated syndromes, termed functional and non-functional tumors, respectively [5]. In this case, we focus exclusively on functional insulinomas, specifically those associated with MEN1. Insulinomas are located in the pancreas and secrete insulin, causing a syndrome characterized by symptoms of hypoglycemia [5, 6]. The most common symptoms are those secondary to hypoglycemia affecting the central nervous system (headache, confusion, blurred vision, behavioral changes, coma, etc.) or due to catecholamine excess (diaphoresis, palpitations, tremors, irritability, etc.) [5, 7]. Our patient presented with a clinical picture highly suggestive of an insulinoma, with a history of MEN1 and a functional insulinoma. Surgical resection is recommended due to the risk of severe hypoglycemia, which can lead to long-term neurological damage [6]. There is no single recommended surgical approach for these patients. In our patient, a distal pancreatectomy was performed for the initial tumor in the tail, followed by a Whipple procedure when recurrence occurred in the pancreatic head.

Our patient experienced a recurrence of a malignant insulinoma. Malignant insulinomas are rare, with only 5%–12% of cases reported as malignant [3]. The management of malignant insulinomas varies, as these tumors carry risks of metastasis and severe hypoglycemia. They are considered malignant when there is local tissue invasion, lymph node involvement, microvascular invasion, or distant metastases (e.g. liver) [3]. Only 2% of malignant insulinomas present with distant metastases. Nevertheless, surgical resection remains the first-line treatment [8, 9]. Literature reports that in patients with malignant insulinomas, local or distant recurrence occurs in ~30%–50% of cases during long-term follow-up. This is particularly relevant in the context of MEN1, where the multifocal nature of the tumors increases the risk of recurrence, even after apparently complete surgical resection [3, 8].

Malignant insulinomas in the context of MEN1 are extremely rare but present a clinical challenge due to their high propensity for recurrence, both locally and at distant sites, even after seemingly complete surgical resection. This case underscores the need for long-term follow-up, including clinical and imaging evaluations, to allow for early detection of recurrences and the timely implementation of additional therapeutic strategies.
